# P-943. Adherence to Guideline-Recommended Duration of Antibiotic Treatment for Uncomplicated Cardiovascular Implantable Electronic Device Lead Infections

**DOI:** 10.1093/ofid/ofae631.1134

**Published:** 2025-01-29

**Authors:** Shenae Cummings, Hiba Al Shaikhli, Muhammad R Sohail, Prakhar Vijayvargiya, Gabriel Hernandez, James Bensler, Zerelda Esquer Garrigos

**Affiliations:** University of Mississippi Medical Center, jackson, Mississippi; University of Mississippi Medical Center, jackson, Mississippi; Baylor College of Medicine, Houston, Texas; University of Mississippi Medical Center, jackson, Mississippi; University of Mississippi Medical Centre, Jackson, Mississippi; University of Mississippi Medical Center, jackson, Mississippi; University of Mississippi Medical Center, jackson, Mississippi

## Abstract

**Background:**

Cardiovascular Implantable Electronic Device lead infection (CIEDLI) is defined as infection involving the leads without concurrent valvular endocardial involvement. These infections are categorized as complicated when extracardiac infection is present and as uncomplicated when distant sites of infection are absent. According to the American Heart Association (AHA), uncomplicated CIEDLI due to Staphylococcus aureus should be treated for 2 to 4 weeks of antibiotic treatment, while non-S. aureus infections typically require a 2-week course. We aimed to evaluate physician adherence to AHA guidelines regarding duration of treatment for CIEDLI.

**Methods:**

We retrospectively reviewed adults presenting with CIEDLI at our institution between 2015 and 2023 to identify those with uncomplicated infection. Physician recommendations regarding treatment duration for CIEDLI were examined. Patients with evidence of CIED-related endocarditis, complicated CIEDLI, and pocket infections without evidence of CIEDLI were excluded.

**Results:**

Twenty-three patients met study criteria. The median age at the time of presentation was 66 years (IQR 46-75) with the majority being male (13/23, 57%) and Caucasian (12/23, 52%). Most patients had an Implantable Cardioverter Defibrillator (11/23, 48%). S. aureus was the most common causative pathogen (13/23, 56%), followed by coagulase negative staphylococci. Only 30% (7/23) of patients received therapy for the duration recommended by guidelines, while the remaining 70% were treated for longer periods, up to six weeks, despite having uncomplicated infection. Side effects to antibiotic therapy (acute kidney injury) were reported in two patients receiving prolonged treatment, whereas none were reported in the group treated according to guidelines.

**Conclusion:**

Our study highlights a lack of adherence to AHA guidelines regarding the duration of antibiotic treatment for uncomplicated CIEDLI. Future efforts should focus on improving awareness and implementing evidence-based practices to optimize patient outcomes, minimize unnecessary antibiotic exposure, potentially mitigating the development of antibiotic resistance, and conserve healthcare resources.

**Disclosures:**

**Muhammad R. Sohail, MD**, Angiodynamics: consulting fees from Medtronic, Phillips and Angiodynamics|Medtronic: Honoraria|Medtronic: Consulting fees from Medtronic, Phillips and Angiodynamics|Phillips: consulting fees from Medtronic, Phillips and Angiodynamics

